# Targeting Oxidative Stress and Neuroinflammation: Epigallocatechin-3-gallate-Selenium Nanoparticles Mitigate Sleep Deprivation-Induced Cortical Impairment

**DOI:** 10.3390/ijms262211173

**Published:** 2025-11-19

**Authors:** Radwa Hussein Lutfy, Ahmed M. Ashour, Ali Khames, Alzahraa A. Elhemiely, Khaled M. Alam-ElDein, Ahmed Hassan Ibrahim Faraag, Mariam O. A. Hamed, Zainab J. Abdel Daim, Nagwa Ibrahim Attia, Mohamed H. A. Gadelmawla

**Affiliations:** 1 Medical Biotechnology Department, School of Biotechnology, Badr University in Cairo, Cairo 11829, Egypt; radwa.hussien_pg@alexu.edu.eg (R.H.L.); professor_ahmed85@science.helwan.edu.eg (A.H.I.F.); mariam.osama@buc.edu.eg (M.O.A.H.); 2 Department of Pharmacology and Toxicology, College of Pharmacy, Umm Al-Qura University, Makkah 21955, Saudi Arabia; amashour@uqu.edu.sa; 3 Department of Pharmacology and Toxicology, Faculty of Pharmacy, Sohag University, Sohag 82511, Egypt; ali.khames@pharm.sohag.edu.eg; 4 Department of Pharmacology, Egyptian Drug Authority (EDA)—Formerly National Organization for Drug Control and Research (NODCAR), Giza 12654, Egypt; alzahraaelhemiely@gmail.com; 5 Botany and Microbiology Department, Faculty of Science, Helwan University, Ain Helwan, Cairo 11795, Egypt; 6 Biology Department, College of Science, Jazan University, Jazan 82817, Saudi Arabia; zdayem@jazanu.edu.sa; 7 Department of Neurology, Faculty of Medicine, Zagazig University, Zagazig 44519, Egypt; dr.nagwa89neuro@gmail.com; 8 Department of Life Sciences, Faculty of Biotechnology, Sinai University, Kantara Branch, Ismailia 41636, Egypt

**Keywords:** sleep deprivation, inflammation, neurotransmitter, Nrf2, cerebral cortex, oxidative stress, neurotropic, glial

## Abstract

Sleep deprivation (SD) has been revealed to provoke anxiety-like behavior. Phytochemicals and nanotechnology-based interventions have emerged as promising alternatives due to their pleiotropic activity and enhanced bioavailability. Here we investigated the effect of sodium selenite, Epigallocatechin-3-gallate (EGCG), and EGCG–Selenium nanoparticles (SeNPs) on SD-provoked cortical impairment and tried to recognize the possible underlying mechanisms in addition to in silico analysis of EGCG. SD was provoked in rats utilizing a modified multiple platform model. We performed an in silico analysis of EGCG docked on Bcl2 and MMP2. Forty animals were divided into five groups of eight animals each: animals were given saline orally for 8 days (control); animals were given saline orally for 8 days, and on day 7 animals were exposed to 24 h of SD (24 h SD); animals were given Na_2_SeO_3_ orally with 0.5 mg/kg/day for 8 days, and on day 7 animals were exposed to 24 h of SD (24 h SD/Na_2_SeO_3_); animals were given 100 mg/kg/day EGCG orally for 8 days, and on day 7 animals were exposed to 24 h of SD (24 h SD/EGCG); animals were given SeNPs biosynthesized using EGCG and 0.5 mg/kg/day orally for 8 days, and on day 7 animals were exposed to 24 h of SD (24 h SD/EGCG-SeNPs). Behavioral tests were performed, including the sucrose preference test and the open-field test. Neurotransmitters (norepinephrine, serotonin, and dopamine), monoamine oxidase, ACh, GABA, AChE, neurotropic and glial markers (BDNF and GFAP), as well as neuro-inflammatory, oxidative stress, and apoptotic markers were assessed. Interestingly, sodium selenite, EGCG, and EGCG-SeNP employment mitigated cognitive functions and cortical histopathological alterations in SD-subjected rats. These potential impacts elicited by sodium selenite, EGCG, and EGCG-SeNPs may be related to their impact of elucidating corticosterone increase, cortical neurotransmitter decrease, and neurotropic and glial markers alterations, while also inhibiting the inflammatory and apoptotic axis and upregulating Nrf2 antioxidant cascade. These results prove the neuroprotective potential of sodium selenite, EGCG, and EGCG-SeNPs, especially EGCG-SeNPs in sleep deprivation-subjected rats by ameliorating cortical neuroinflammation, prooxidant alterations, and apoptotic events likely caused by modulating the NOS-2/Nrf2 axis.

## 1. Introduction

Sleep deprivation (SD) is a pervasive modern stressor with profound consequences on brain health, behavior, and overall physiology. Acute or chronic loss of sleep is closely linked to cognitive deficits, impaired memory consolidation, mood dysregulation, and heightened vulnerability to neurodegenerative disorders [1]. Mechanistically, SD induces a cascade of pathological processes, including oxidative stress, neuroinflammation, apoptosis, neurotransmitter imbalance, and neuroendocrine dysfunction, which together drive structural and functional brain alterations [2]. Despite its clinical relevance, therapeutic strategies that effectively mitigate the neurotoxic impact of SD remain limited, warranting exploration of novel, multi-targeted interventions [3].

Oxidative stress is considered a central mediator of SD-induced neuropathology. Elevated levels of lipid peroxidation products such as MDA and the reactive free radical NO are consistently reported in SD models, indicating enhanced oxidative burden [4]. This redox imbalance is further aggravated by the depletion of endogenous antioxidants, including GSH, and the reduced activity of enzymatic scavengers such as SOD and CAT [5]. Transcriptional dysregulation of the antioxidant defense regulator Nrf2 also contributes to diminished cellular resilience, promoting oxidative damage to neuronal membranes, proteins, and DNA [6].

Parallel to oxidative stress, neuroinflammation plays a pivotal role in SD-related brain injury. Pro-inflammatory cytokines such as TNF-α, IL-6, and IL-1β are markedly elevated in experimental SD, while inducible nitric oxide synthase (NOS2) activation exacerbates nitrosative stress [7]. This inflammatory milieu facilitates microglial activation and amplifies neuronal damage, impairing synaptic integrity and cognitive performance [8].

Apoptotic signaling further accelerates neurodegeneration under SD. Increased production of pro-apoptotic proteins Bax and caspase-3, together with suppression of the anti-apoptotic marker Bcl-2, has been shown in sleep-deprived brains [9]. These molecular alterations contribute to neuronal shrinkage, synaptic loss, and functional decline, particularly in hippocampal and cortical regions that regulate learning, memory, and emotional processing [10].

Disruption of neurotransmitter systems is another defining feature of SD. Monoamines such as serotonin (5-HT), dopamine (DA), and norepinephrine (NE) are depleted, contributing to impaired mood regulation, arousal, and cognitive flexibility. In parallel, cholinergic dysfunction is evident, with reduced acetylcholine (ACh) levels and increased activity of acetylcholinesterase (AChE) and monoamine oxidase (MAO), which are enzymes responsible for neurotransmitter degradation [11]. Moreover, altered expression of glutamatergic receptors, such as Grin1, further destabilizes excitatory–inhibitory balance, leading to excitotoxicity and synaptic failure [12,13].

Neurotrophic and glial markers also undergo marked alterations. BDNF, a critical regulator of synaptic plasticity, neuronal survival, and memory processes, is downregulated following SD, correlating with impaired cognitive and affective outcomes [14]. At the same time, elevated expression of GFAP reflects reactive astrogliosis and glial activation, which intensify neuro-inflammatory cascades and reduce neurotrophic support [15].

Neuroendocrine response to SD is characterized by hyperactivation of the hypothalamic–pituitary–adrenal (HPA) axis [16,17]. Increased corticosterone levels in rodents, analogous to cortisol in humans, serve as a biochemical hallmark of sleep loss-induced stress [18]. Sustained corticosterone elevation is associated with neuronal atrophy, reduced neurogenesis, and exacerbation of oxidative and inflammatory injury within limbic brain regions [19].

Given the multifactorial pathogenesis of SD-induced neurotoxicity, therapeutic approaches targeting a single pathway are often inadequate. Phytochemicals and nanotechnology-based interventions have emerged as promising alternatives due to their pleiotropic activity and enhanced bioavailability [20]. Epigallocatechin-3-gallate (EGCG), the principal catechin in green tea, has potent antioxidant, anti-inflammatory, and neuroprotective properties, including the ability to cross the blood–brain barrier, scavenge reactive species, inhibit prooxidant enzymes, and upregulate BDNF expression [21,22].

Selenium, a vital trace element with recognized roles in redox homeostasis and neuroprotection, has likewise demonstrated efficacy in mitigating oxidative and inflammatory injury [23]. Importantly, the biosynthesis of selenium nanoparticles (SeNPs) using EGCG not only exploits the reducing and stabilizing capacities of EGCG but also confers synergistic neuroprotective properties [24]. These EGCG-SeNPs exhibit superior bioavailability, controlled release, and enhanced antioxidant, anti-inflammatory, and anti-apoptotic activity compared to either agent alone [25,26].

Based on these observations, we hypothesized that EGCG-mediated SeNPs could provide a robust therapeutic strategy against SD-induced neuropathology. By simultaneously modulating oxidative stress, inflammatory mediators, apoptotic signaling, neurotransmitter dynamics, neurotrophic and glial markers, and corticosterone levels, EGCG-SeNPs hold the potential to restore neuronal homeostasis and mitigate the deleterious consequences of sleep deprivation.

## 2. Results

### 2.1. In Silico Analysis

#### 2.1.1. Docking Score

**Figure 1:**
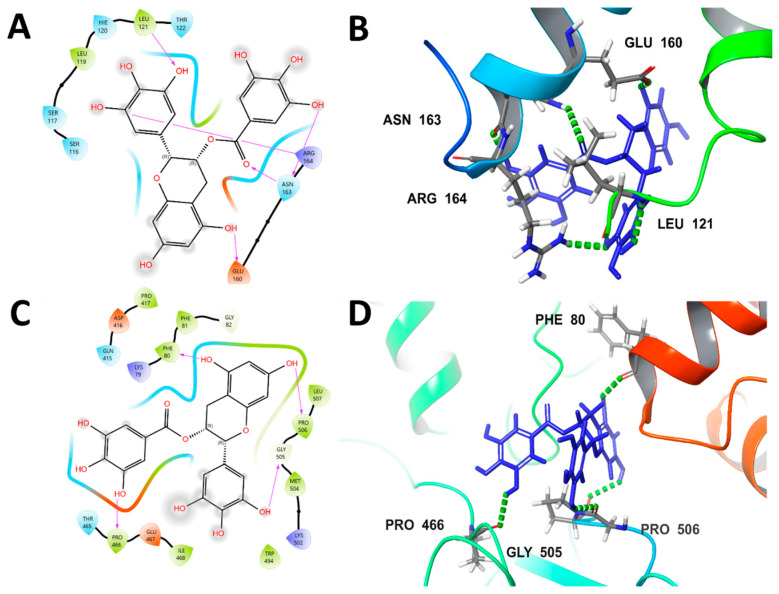
Molecular docking visualization of EGCG binding interactions. (**A**) 2D interaction and (**B**) 3D interactions of anti-apoptotic BCL-2 and (**C**) 2D interaction and (**D**) 3D interactions of Metalloproteinase MMP-2.

Molecular docking analysis revealed that epigallocatechin gallate (EGCG) exhibited favorable binding interactions with both anti-apoptotic BCL-2 and metalloproteinase MMP-2. The Glide XP G-scores were −4.033 kcal mol^−1^ for BCL-2 and −6.749 kcal mol^−1^ for MMP-2, indicating good and high binding affinities, respectively. EGCG formed specific hydrogen bonds with key residues in the BCL-2 active site (Leu 121, Glu 160, Asn 163, and Arg 164) and established hydrogen-bonding interactions with critical residues in the MMP-2 binding pocket (Phe 80, Pro 466, Gly 505, and Pro 506). These findings demonstrate EGCG’s strong molecular recognition and potential dual inhibitory activity against both therapeutic targets (Table 1 and Figure 1).

#### 2.1.2. Absorptivity Measurement of Nanomaterials

**Figure 2:**
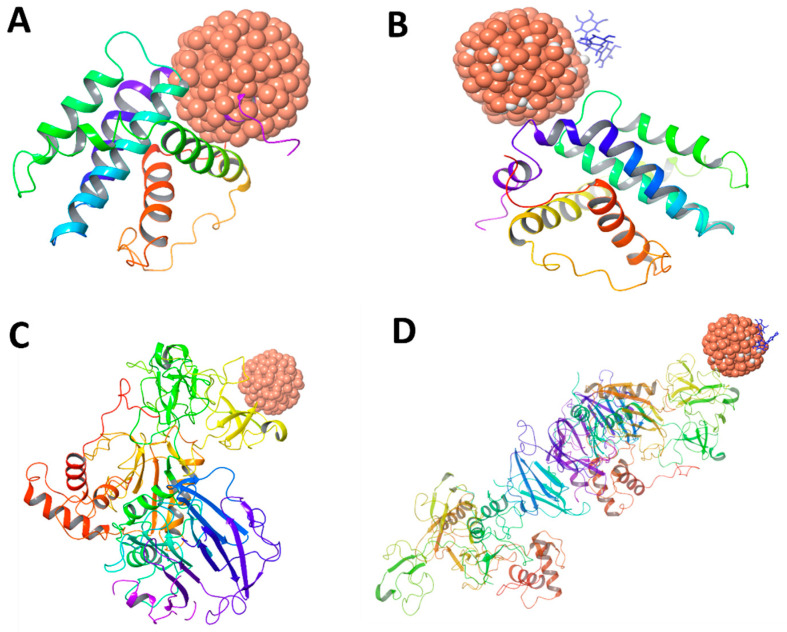
Absorptivity models of BCL2 and MMP2 proteins with SeNPs and EGCG-functionalized SeNPs: (**A**) BCL2-SeNPs, (**B**) BCL2-EGCG-SeNPs, (**C**) MMP2-SeNPs, and (**D**) MMP2-EGCG-SeNPs.

The adsorption properties of nano-selenium and EGCG–nano-selenium with BCL2 and MMP2 structures were investigated using the BIOVIA Materials Studio 2024 software suite. The lowest energy poses were initially determined using the adsorption locator module, which employs Forcite and COMPASS III force fields. The total energy, adsorption energy, rigid adsorption energy, and deformation energy were then calculated for each system. As shown in the results, the BCL2-EGCG–nano-selenium complex exhibited a total energy of 1.21 × 10^5^ and an adsorption energy of −8.16 × 10^4^. The deformation energy for this complex was calculated to be −1.026 × 10^4^. In comparison, the BCL2–nano-selenium system showed a total energy of 2.49 × 10^4^ and an adsorption energy of −5.44 × 10^4^, with a deformation energy of −1.19 × 10^4^. For the MMP2-EGCG–nano-selenium complex, the total energy was 1.36 × 105 and the adsorption energy was −1.01 × 10^5^, with a deformation energy of −3.21 × 10^4^. The MMP2–nano-selenium system had a total energy of 3.64 × 10^4^ and an adsorption energy of −7.69 × 10^4^, with a deformation energy of −3.62 × 10^4^. The rigid adsorption energy values for the complexes were also determined, ranging from −22.15 to 6.015. The results indicate that the EGCG–nano-selenium ligand consistently exhibited more negative adsorption energies compared to the nano-selenium ligand alone when interacting with both BCL2 and MMP2, suggesting a more favorable and stable binding interaction (Table 2 and Figure 2).

### 2.2. SeNPs-EGCG Characterization

#### 2.2.1. Transmission Electron Microscope (TEM), Zeta Size, and Zeta Potential

**Figure 3:**
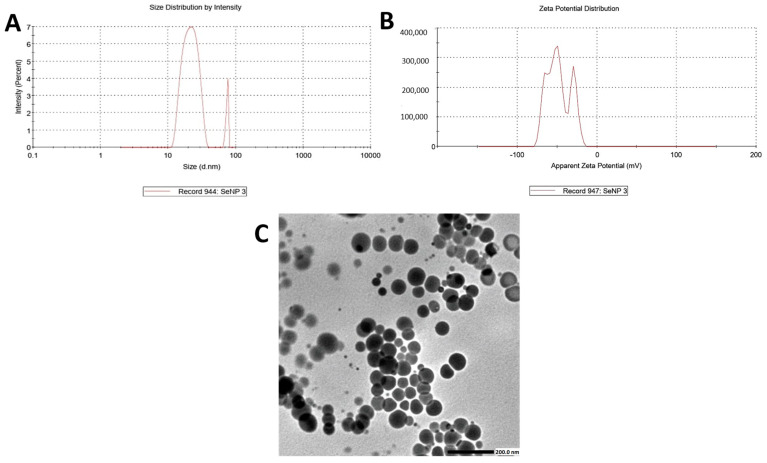
(**A**) Zeta size, (**B**) zeta potential, and (**C**) TEM of selenium nanoparticles showing successful preparation (Scale bar = 200 nm).

Transmission electron microscopy (TEM) analysis revealed that the EGCG-assisted biosynthesis of selenium nanoparticles (SeNPs) yielded predominantly spherical structures with a relatively uniform morphology. The particle sizes were mainly distributed within the 30–60 nm range, consistent with nanoscale dimensions (Figure 3). While the nanoparticles exhibited adequate dispersion across several regions, partial aggregation was also observed, likely attributable to the intrinsic high surface energy of SeNPs, even in the presence of EGCG functioning as a dual reducing and stabilizing agent. These results show the successful green synthesis of SeNPs, characterized by controlled geometry, narrow size distribution, and stable nanoscale features.

#### 2.2.2. UV–Vis Spectroscopic Characterization

**Figure 4:**
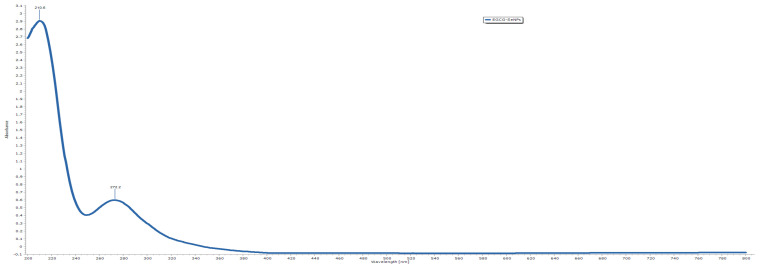
UV–vis result of selenium nanoparticles, showing successful preparation.

The optical absorption spectrum of the biosynthesized EGCG-SeNPs exhibited a prominent absorption peak at 210.6 nm, accompanied by a secondary band at 272.2 nm (Figure 4). The sharp peak at 210.6 nm corresponds to the π → π* transition of aromatic rings within EGCG, while the minor band near 272.2 nm reflects the n → π* transition associated with phenolic functional groups, confirming the interaction of EGCG molecules with selenium ions. The absence of additional peaks beyond 300 nm and the gradual decline in absorbance across the visible region indicate the formation of stable, well-dispersed SeNPs without aggregation.

#### 2.2.3. FT-IR Analysis

The FTIR analysis was conducted to confirm the molecular structure of EGCG and to elucidate the nature of the interaction between EGCG and selenium in the nanocomposite. The spectra for both pure EGCG and the EGCG-SeNPs complex display several characteristic peaks corresponding to the major functional groups present in the EGCG molecule. A comparison of the two spectra reveals distinct shifts in peak positions and changes in intensity, providing evidence for the successful formation of the complex.

##### FTIR Peaks of Pure EGCG

The FTIR spectrum of pure EGCG shows its characteristic functional group peaks (Table 3). A broad and strong absorption band observed at 3241 cm^−1^ is attributed to the O-H stretching vibration, arising from the multiple phenolic hydroxyl groups present in the EGCG structure. The broadness of this peak is indicative of strong intermolecular and intramolecular hydrogen bonding. The peaks at 2918 cm^−1^ and 2849 cm^−1^ correspond to the C-H stretching vibrations of the aliphatic -CH_2_ and -CH_3_ groups within the flavan-3-ol backbone. A prominent and sharp peak at 1704 cm^−1^ is assigned to the C=O stretching vibration of the ester carbonyl group in the gallate moiety of EGCG. The region between 1600 and 1450 cm^−1^, with peaks at 1610 cm^−1^ and 1448 cm^−1^, is associated with the aromatic C=C stretching vibrations of the benzene rings. Furthermore, the peaks observed at 1242 cm^−1^ and 1053 cm^−1^ are characteristic of the C-O stretching vibrations from the phenolic and ester groups, respectively. These peaks collectively confirm the chemical structure of the EGCG sample.

##### FTIR Peaks and Interaction in the EGCG-SeNPs Complex

**Figure 5:**
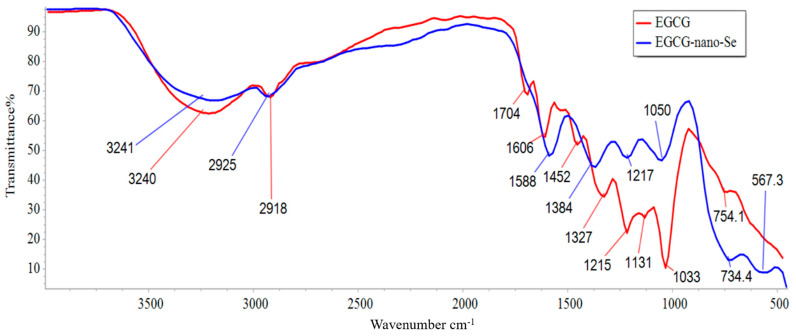
FTIR peaks between EGCG and EGCG-SeNPs.

The FTIR spectrum of the EGCG–nano-Se complex shows significant changes compared to that of pure EGCG, confirming the interaction between EGCG and the selenium nanoparticles (Table 4). The most notable change is observed in the broad O-H stretching band. In the complex, this band shifts from 3241 cm^−1^ to a lower wavenumber of approximately 3225 cm^−1^ and becomes broader. This red-shift and broadening suggest that the phenolic hydroxyl groups of EGCG are involved in the coordination with the nano-selenium surface, likely through the formation of Se-O bonds or enhanced hydrogen bonding, which weakens the O-H bond. Similarly, the C=O stretching vibration of the ester group shifts from 1704 cm^−1^ to 1698 cm^−1^, indicating that the carbonyl oxygen may also participate in the stabilization of the nanocomposite. The peaks corresponding to C-O stretching vibrations at 1242 cm^−1^ and 1053 cm^−1^ also shift to lower wavenumbers (e.g., to 1235 cm^−1^ and 1048 cm^−1^), further supporting the involvement of oxygen-containing groups in the interaction. The aromatic C=C stretching peaks show minor shifts, suggesting that the core aromatic structure remains largely intact, but the electronic environment is altered due to the complex formation. Overall, the observed shifts in the key functional group peaks provide strong spectroscopic evidence that EGCG successfully acts as a capping and reducing agent, binding to the surface of selenium nanoparticles through its hydroxyl and carbonyl groups (Figure 5).

### 2.3. Sodium Selenite, EGCG, and EGCG-SeNPs Enhance the Performance of Sleep Deprivation-Subjected Rats in the Open-Field Test

Three tests were applied to evaluate the impact of sodium selenite, EGCG, and EGCG-SeNPs on depression-like behavior using the OFT. The results from these tests revealed that sodium selenite, EGCG, and EGCG-SeNPs improved sucrose preference, rearing and grooming time, and alleviated anhedonic-like behavior in sleep deprivation-subjected rats.

**Figure 6:**
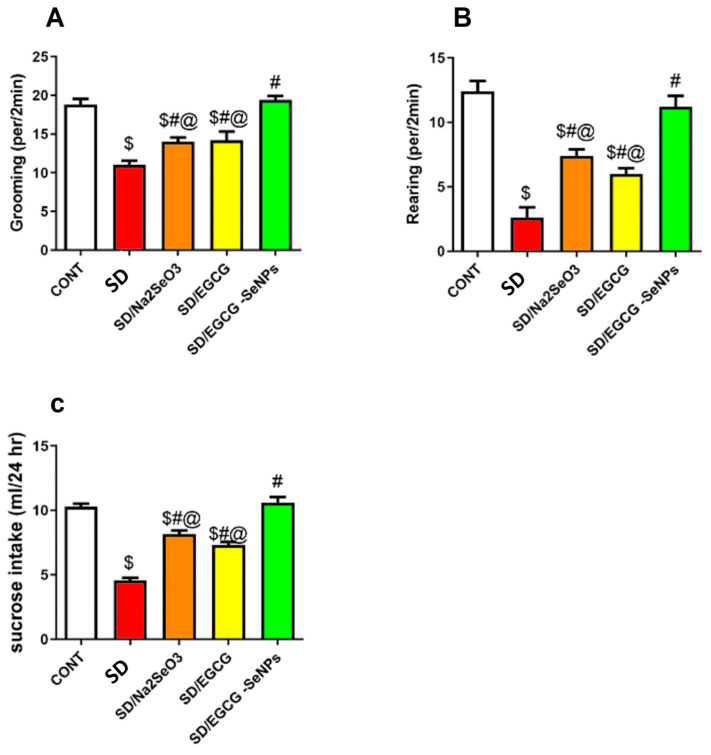
Effect of selenium selenite, EGCG, and EGCG-SeNPs on (**A**) grooming, (**B**) rearing, and (**C**) sucrose intake in sleep deprivation-subjected rats (N = 8). Data represented as mean ± SEM; one way analysis of variance (ANOVA) was performed, followed by Tukey’s multiple comparison post hoc test to evaluate intergroup differences. $ Significant to the normal control group; # significant to sleep deprivation group; @ significant to EGCG-SeNPs.

As illustrated in (Figure 6), SD caused a remarkable reduction in rearing, grooming, and sucrose-intake frequencies as compared with the negative control group. The co- treatment with sodium selenite, EGCG, and EGCG-SeNPs significantly increased rearing and grooming frequencies and sucrose intake, in comparison with the sleep deprivation-subjected rats. For more accuracy, we found that EGCG-SeNPs markedly raised the rearing and grooming frequencies, as well as sucrose intake, versus the sodium selenite- and EGCG-co-treated group. Additionally, EGCG-SeNPs increased rearing and grooming frequencies, making them similar to those of the control animals as there was no remarkable variance between the EGCG-SeNP-co-treated group and normal control group.

### 2.4. The Effects of EGCG-SeNPs on Brain Monoamine Contents in the Sleep Deprivation-Subjected Rats

**Figure 7:**
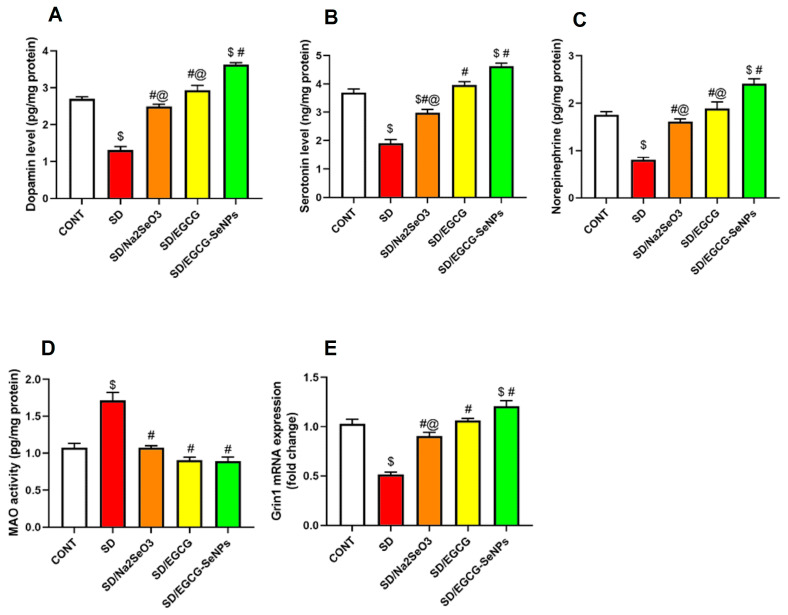
Impact of selenium selenite, EGCG, and EGCG-SeNPs on (**A**) dopamine, (**B**) serotonin, (**C**) norepinephrine, (**D**) MAO, and (**E**) Grin 1 in sleep deprivation-challenged rats. In each group, *n* = 8 and data are represented as mean ± SEM; one way analysis of variance (ANOVA) was applied, followed by Tukey’s multiple comparison post hoc test to evaluate intergroup differences. $ Significant to the normal control group; # significant to sleep deprivation group; @ significant to EGCG-SeNPs.

As represented in Figure 7, there were significant differences in cortical dopamine, serotonin, norepinephrine, and Grin 1 gene expression between different groups. Sleep deprivation significantly decreased dopamine, serotonin, norepinephrine, and Grin 1 gene expression, along with considerable increases in monoamine oxidase activity in the prefrontal cortex as compared to the normal control rats. Indeed, co-treatment with sodium selenite, EGCG, and EGCG-SeNPs significantly increased dopamine, serotonin, norepinephrine, and Grin 1 gene expression with significant decline in monoamine oxidase activity in the prefrontal cortex as compared with the sleep-deprived-rats. Moreover, EGCG-SeNP co-administration markedly elevated dopamine and norepinephrine versus sodium selenite- and EGCG-co-treated animals, and upregulated serotonin and Grin 1 gene expression versus sodium selenite-co-treated animals.

Additionally, EGCG-SeNPs significantly increased dopamine, serotonin, norepinephrine, and Grin 1 gene expression in the cortex versus the normal control group.

So, these findings confirm sodium selenite, EGCG, and particularly EGCG-SeNPs’ ability to enhance the aforementioned cortical neurotransmitters, overcoming the depression manifested in sleep deprivation-subjected rats.

### 2.5. EGCG-SeNPs Can Restore AChE Activity, ACh and GABA Levels in Sleep Deprivation-Subjected Rats

**Figure 8:**
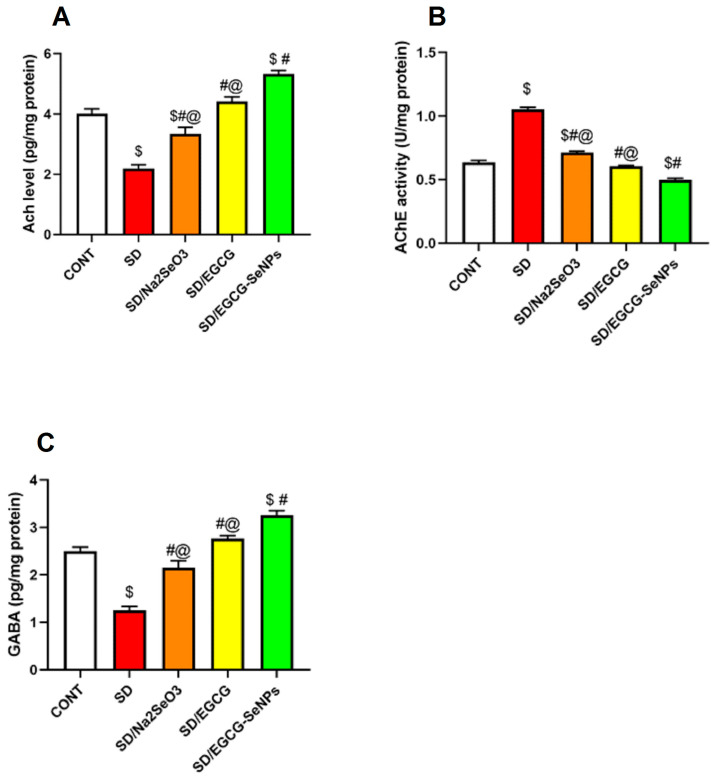
Selenium selenite, EGCG, and EGCG-SeNPs’ effect on (**A**) acetylcholine level, (**B**) acetylcholine esterase activity, and (**C**) γ-aminobutyric acid (GABA) in sleep deprivation-subjected rats. In each group, *n* = 8 and data are represented as mean ± SEM; one way analysis of variance (ANOVA) was performed, followed by Tukey’s multiple comparison post hoc test to evaluate intergroup differences. $ Significant to the normal control group; # significant to sleep deprivation group; @ significant to EGCG-SeNPs.

To investigate the neurotransmitter alterations correlated with sleep deprivation, brain values of acetylcholine and its degrading enzyme acetylcholinesterase were examined. It is known that cholinergic transmission in the cortex is associated with cognitive impairment. Meanwhile, the amount of GABA was determined. Figure 6 shows that the levels of cortical acetylcholine, acetylcholine esterase activity, and GABA differed significantly between groups. Cortical acetylcholine and GABA values were substantially lower than those in the control group; moreover, acetylcholine esterase activity was significantly elevated. By administering sodium selenite, EGCG, and EGCG-SeNPs to sleep deprivation-subjected rats, these neurotransmitter changes were reversed, as detected by a remarkable increase in acetylcholine and GABA which was accompanied by a substantial decline in acetylcholine esterase. Notably, EGCG-SeNP co-treatment exerts significant increase in acetylcholine and GABA which sees a significant decrease in acetylcholine esterase activity when compared to sodium selenite- and EGCG-co-treated rats and normal control rats (Figure 8).

According to these data, sodium selenite, EGCG, and EGCG-SeNPs can improve neurotransmitter changes, so engaging in rescuing the cognitive impairment associated with sleep deprivation is significantly linked to EGCG-SeNPs.

### 2.6. Sodium Selenite, EGCG, and EGCG-SeNPs Can Ameliorate the Cortical Oxidative Stress in the Sleep Deprivation-Subjected Rats

**Figure 9:**
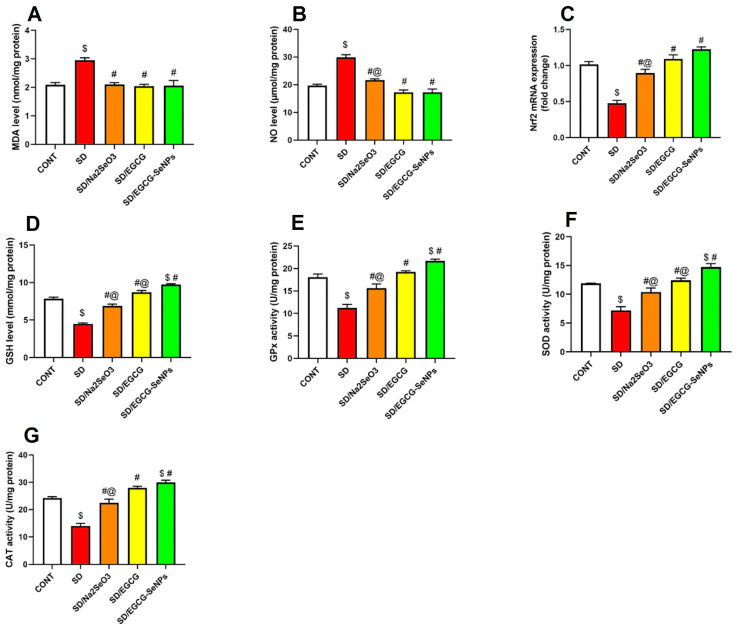
Effect of sodium selenite, EGCG, and EGCG-SeNPs on (**A**) MDA, (**B**) NO, (**C**) Nrf2, (**D**) GSH, (**E**) GPx, (**F**) SOD, and (**G**) CAT in sleep deprivation-subjected rats. Data are represented as mean ± SEM (N = 8). In the analysis, one way analysis of variance (ANOVA) was performed, followed by Tukey’s multiple comparison post hoc tests to evaluate intergroup differences. $ Significant to the normal control group; # significant to sleep deprivation group; @ significant to EGCG-SeNPs.

As seen in Figure 9, sleep deprivation resulted in a remarkable increase in oxidative stress responses in the rat brain. This process was characterized by a significant rise in MDA content and NO values in the prefrontal cortex. Compared to those in the control group, the expression levels of genes implicated in the antioxidant system, including Nrf2 and its downstream effectors GSH, GPx, SOD, and catalase, were significantly reduced.

Compared with the SD group, the administration of sodium selenite, EGCG, and EGCG-SeNPs to rats that were subjected to SD successfully reduced oxidative stress responses, resulting in a remarkable drop in brain MDA content and NO level in the prefrontal cortex. This effect was further validated by the pronounced upregulation of antioxidant Nrf2 gene expression and the subsequently increase in GSH content, as well as GPx, catalase, and SOD activities.

A comparative analysis of the three compounds revealed that EGCG-SeNP administration resulted in superior antioxidative stress effects. Compared with sodium selenite-co-treated rats, it significantly decreased NO and increased Nrf2 gene expression, GPx, and catalase activities. Additionally, it increased GSH content and SOD activity versus the EGCG-co-treated group. Moreover, for GSH content, GPx, catalase, and SOD activities in the EGCG-SeNP co-group revealed a substantial rise compared to the normal control animals.

These findings illuminate the role of sodium selenite, EGCG, and EGCG-SeNPs in mitigating oxidative stress and activating the Nrf2 cascade, contributing at least partially to the amelioration of cognitive impairment induced by SD.

### 2.7. Sodium Selenite, EGCG, and EGCG-SeNPs Can Abrogate Inflammation in the Sleep Deprivation-Subjected Rats

Chronic SD has been found to decrease cognitive performance, affect mood states, and simultaneously dysregulate inflammatory and stress hormones.

**Figure 10:**
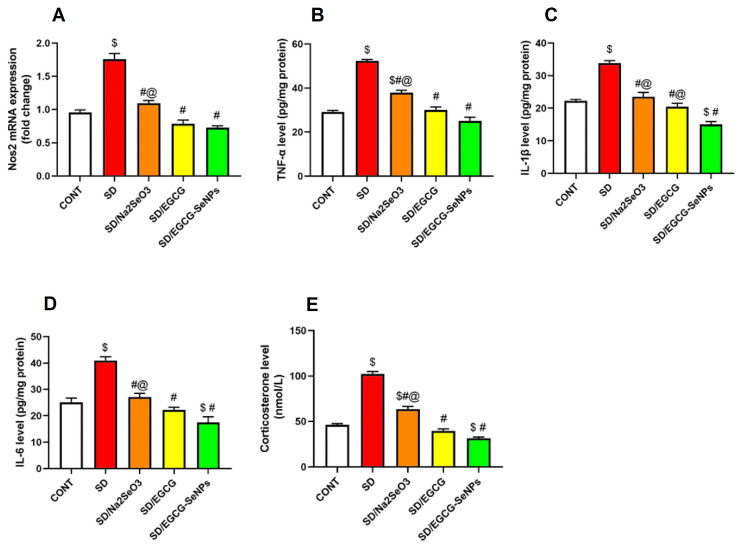
Selenium selenite, EGCG, and EGCG-SeNPs altered (**A**) NOS2 gene expression, (**B**) TNF-α, (**C**) IL-1β, (**D**) IL-6, and (**E**) corticosterone in sleep deprivation-subjected rats. In each group, *n* = 8 and data are represented as mean ± SEM; one way analysis of variance (ANOVA) was performed, followed by Tukey’s multiple comparison post hoc test to evaluate intergroup differences. $ Significant to the normal control group; # significant to sleep deprivation group; @ significant to EGCG-SeNPs.

In our work, mRNA expression of NOS2 showed a rising trend in the prefrontal cortex of the SD group compared to the normal group. However, versus the sleep deprivation group, sodium selenite, EGCG, and EGCG-SeNPs showed a tendency to decline the mRNA expression of NOS2 (Figure 10). In the same context, TNF-α, IL-1β, and IL-6 levels remarkably elevated in the prefrontal cortex of the SD group, compared to the normal group. A marked decrease in TNF-α, IL-1β, and IL-6 levels was observed with the sodium selenite-, EGCG-, and EGCG-SeNP-co-treated groups compared to the SD group. Additionally, the EGCG-SeNP-co-treated group exerts more powerful anti-inflammatory action that exceeds that of the normal control rats and other co-treated groups as it considerably downregulated NOS2, TNF-α, and IL-6, compared to the sodium selenite-co-treated group, and decreased IL-1β versus the sodium selenite- and EGCG-co-treated group. And additionally, it significantly decreased IL-1β and IL-6 compared to the normal control group.

Regarding corticosterone level, stress factor was substantially elevated in the SD group compared to the normal control group. Co-administration of sodium selenite, EGCG, and EGCG-SeNPs resulted in a remarkable decrease in corticosterone level compared to the sleep deprivation group, confirming the ability to attenuate the stress response.

Considering the comparison between treated groups, EGCG-SeNPs decrease corticosterone level significantly versus the normal control and the sodium selenite-co-administration groups.

**Figure 11:**
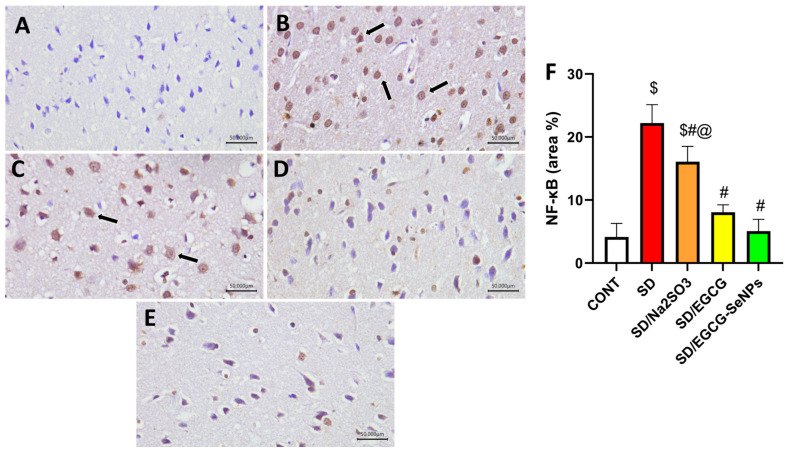
Protective effect of EGCG-SeNPs on prefrontal cortex immunoreactivity of NF-κB in SD-evoked cortical damage in rats. Photomicrographs of the prefrontal cortex from all groups. The immunoreactivity of NF-κB were visible in the tissues as a brown color generated by DAB chromogen (arrow) (DAB, X400, Scale bar = 50 µm). The control group (**A**) exhibited normal cortical structure with weak GFAP staining. SD (**B**) and SD–sodium-selenite-treated (**C**) groups revealed intense NF-κB immunostaining. In contrast, the (**D**) SD-EGCG- and (**E**) SD-EGCG-SeNP-treated group displayed attenuated NF-κB staining. (**F**) Quantitative analysis of immunostaining area % for NF-κB was expressed as mean ± S.E.M. (*n* = 8). Data are represented as mean ± SEM, performed using one way analysis of variance (ANOVA), followed by Tukey’s multiple comparison post hoc test to evaluate intergroup differences. $ Significant to the normal control group; # significant to sleep deprivation group; @ significant to EGCG-SeNPs.

For more accuracy, the transcriptional factor, NF-κB, was estimated by immunohistochemistry. The control rats, EGCG- and EGCG-SeNP-co-treated animals, expressed weak reactivity to NF-κB, contributing to evidence of its anti-inflammatory effect, whereas the sleep deprivation- and sodium selenite-co-treated groups displayed a marked reactivity to NF-κB, indicating inflammatory process. Both EGCG- and EGCG-SeNP-co-treated rats revealed marked decrease in NF-κB compared to the SD group (*p* < 0.0001). However, both SD and SD/sodium selenite groups showed higher NF-κB expression compared to the control group (*p* < 0.0001) (Figure 11). Additionally, administration of EGCG-SeNPs to sleep-deprived-rats decreased NF-κB protein expression significantly versus the sodium selenite-co-treated groups.

### 2.8. Selenium Selenite, EGCG, and EGCG-SeNPs Attenuate Apoptosis in the Sleep Deprivation-Subjected Rats

**Figure 12:**
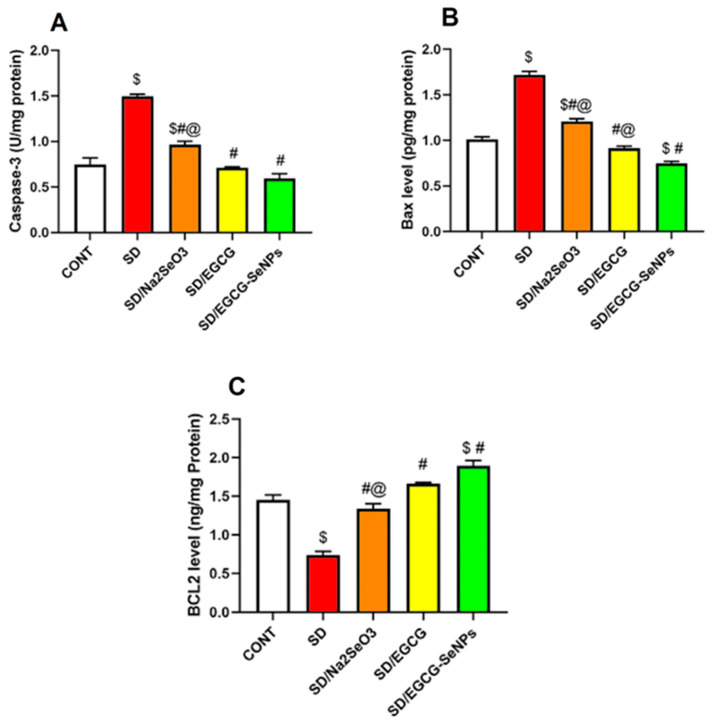
Effect of selenium selenite, EGCG, and EGCG-SeNPs on (**A**) Caspase 3 activity, (**B**) BAX level, and (**C**) BCL2 level in sleep deprivation-subjected rats. In each group, *n* = 8 and data are represented as mean ± SEM; one way analysis of variance (ANOVA) was performed, followed by Tukey’s multiple comparison post hoc test to evaluate intergroup differences. $ Significant to the normal control group; # significant to sleep deprivation group; @ significant to EGCG-SeNPs.

It is believed that cortical apoptotic cell death is correlated with marked degeneration and behavioral deficits in SD-subjected rats. So, the executioner caspase 3, Bax, and Bcl-2 were estimated to determine the cortical apoptosis. As illustrated in Figure 12, it was demonstrated that the rats subjected to sleep deprivation revealed a remarkable elevation in cortical apoptotic markers, caspase-3 activity, and BAX level, together with a considerable decrease in the anti-apoptotic BCL2 level in the prefrontal cortex as compared to the negative control group. On the contrary, sodium selenite-, EGCG-, and EGCG-SeNP-co-treated groups revealed a substantial decline in cortical caspase-3 activity and BAX level, with significant elevation in BCL2 level in the prefrontal cortex versus the sleep deprivation group. Moreover, the EGCG-SeNP-co-treated group markedly decreased in cortical caspase-3 activity and BAX level, with a significant increase in BCL2 level in the prefrontal cortex in comparison with the sodium selenite-co-treated group and considerable decreases in BAX level versus the EGCG-co-treated animals.

Also, for the EGCG-SeNP-co-treated group, it causes a remarkable decrease in BAX level and a significant increase in BCL2, in comparison with the normal control group, confirming its anti-apoptotic effect.

So, depending on these results, sodium selenite, EGCG, and EGCG-SeNPs can curtail apoptotic cell death, especially for the EGCG-SeNP-co-treated group, and abrogate the cognitive decline associated with sleep deprivation.

### 2.9. Selenium Selenite, EGCG, and EGCG-SeNPs Modified the GFAP and BDNF Contents in the Cortical Tissues of the Sleep Deprivation-Subjected Rats

GFAP gene expression has received a lot of interest because its onset is a signal for astrocyte formation and its upregulation indicates reactive gliosis. However, BDNF is the most abundant neurotrophin in the brain, playing a crucial role in the survival, differentiation, synaptic plasticity, and axonal expansion of peripheral and central neurons.

**Figure 13:**
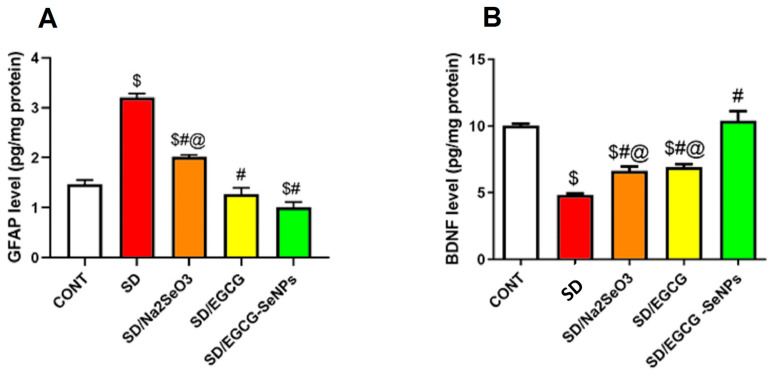
Effect of selenium selenite, EGCG, and EGCG-SeNPs on (**A**) GFAP and (**B**) BDNF levels in sleep deprivation-subjected rats. (*n* = 8) and data are represented as mean ± SEM; one way analysis of variance (ANOVA) was performed, followed by Tukey’s multiple comparison post hoc test to evaluate intergroup differences. $ Significant to the normal control group; # significant to sleep deprivation group; @ significant to EGCG-SeNPs.

As shown in Figure 13, there is a significant rise in the GFAP (a marker of astrocytosis) with marked decrease in BDNF in the prefrontal cortex in the sleep deprivation-subjected group as opposed to the control one. In contrast, sodium selenite-, EGCG-, and EGCG-SeNP-co-treated groups significantly lowered GFAP and upregulated BDNF in comparison to the sleep deprivation group. In the same context, EGCG-SeNPs produce a more favorable effect regarding BDNF compared to sodium selenite and EGCG, and additionally reduce GFAP more than sodium selenite and normal control groups.

**Figure 14:**
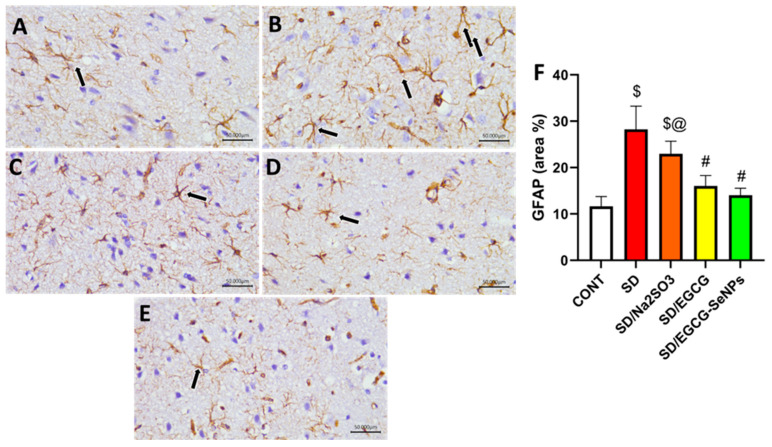
Protective effect of EGCG-SeNPs on prefrontal cortex immunoreactivity of GFAP in SD-evoked cortical damage in rats. Photomicrographs of prefrontal cortex from all groups. The immunoreactivity of GFAP was visible in the tissues as a brown color generated by DAB chromogen (arrow) (DAB, X400, Scale bar = 50 µm). The control group (**A**) exhibited normal cortical structure with moderate GFAP staining. SD- (**B**) and SD–sodium selenite-treated (**C**) groups revealed intense GFAP immunostaining. In contrast, SD-EGCG- (**D**) and SD-EGCG-SeNP-treated (**E**) group displayed attenuated GFAP staining. (**F**) Quantitative analysis of immunostaining area % for GFAP was expressed as mean ± S.E.M. (*n* = 8). Data are represented as mean ± SEM performed using one way analysis of variance (ANOVA), followed by Tukey’s multiple comparison post hoc test to evaluate intergroup differences. $ Significant to the normal control group; # significant to the sleep deprivation group; @ significant to EGCG-SeNPs.

Regarding GFAP immunoreactivity, the control group and EGCG- and EGCG-SeNP-co-treated animals expressed GFAP moderately, which reveals the normal functional glial cellular activity, while the SD group and sodium selenite-co-treated group displayed a marked reactivity to GFAP. Co-administration of EGCG and EGCG-SeNPs produced a considerable reduction in GFAP compared to the SD group (*p* < 0.0001). In contrast, both SD and SD/sodium selenite groups showed higher GFAP expression compared to the control group (*p* < 0.0001) (Figure 14).

### 2.10. Histopathological Examination

**Figure 15:**
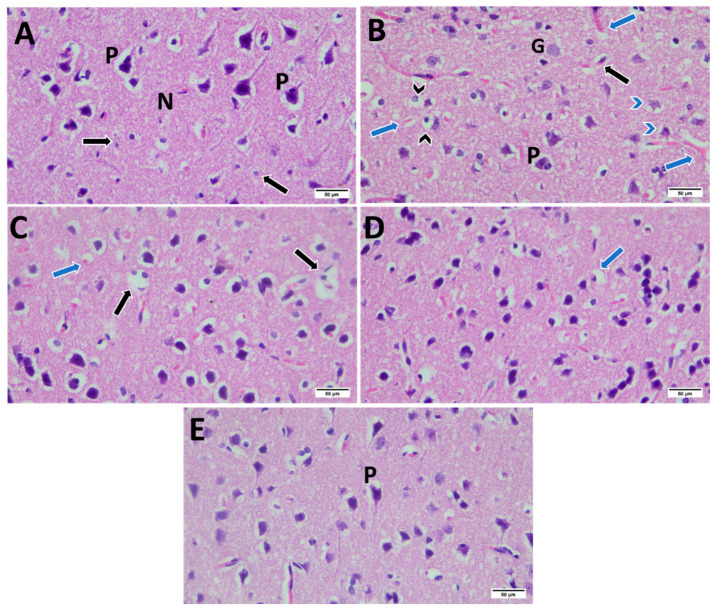
Photomicrographs of male prefrontal cerebral cortex of all groups. (**A**) Control group showed normal neuropil (N) containing vesicular pyramidal cells (P) and multiple neuroglial cells (black arrow). (**B**) 24hSD revealed disturbed cortical architecture with congested blood capillaries (blue arrow), deformed neurons with darkly stained nuclei surrounded by halos, and increased perinuclear space (black arrow head), degenerated pyramidal cells (blue arrow head), and increased neuroglial cells (black arrow). (**C**) 24hSD-Na_2_SeO_3_ showed degenerated cells with pyknotic nuclei (black arrow) and congested capillaries (black arrow). (**D**) 24hSD-EGCG exhibited normal neuropil with few shrunken cells with pyknotic nuclei (arrow). (**E**) 24hSD-EGCG-SeNPs showed approximately normal appearance of cortical tissue with normal pyramidal cells (P). (H&E, X400, Scale bar = 50 µm).

Sections of the prefrontal cortex taken from male control rat displayed normal anatomy. The control group showed granule cells with big, conspicuous nuclei and spherical cell bodies were visible in the outer granular layer. Medium-sized pyramidal cells with basophilic cytoplasm, rounded nuclei, triangular cell bodies, and apical dendrites were visible. The size of the pyramidal cells varied. The neuropil, which was a fibrillar eosinophilic substance, composed of a dense network of branching processes, including glial cells. 24hSD revealed altered architecture of the cerebral cortex, with numerous glial cells visible in the molecular layer. The blood capillaries were dilated, congested, and encircled by perivascular space, degenerated pyramidal cells, and deformed neurons with darkly stained nuclei and surrounded by halos and an increase in the perinuclear space. 24hSD-Na_2_SeO_3_ showed degenerated cells with pyknotic nuclei, congested capillaries, and few deformed neurons, as well as few neurons surrounded by clear areas. 24hSD-EGCG exhibited normal neuropil with few shrunken cells with pyknotic nuclei. 24hSD-EGCG-SeNPs showed approximately normal appearance of cortical tissue with normal neurons and neuropil (Figure 15).

## 3. Discussion

EGCG, a major component of green tea, exhibits promising dual inhibitory activity against BCL-2 and MMP-2, as suggested by molecular docking studies [27,28,29,30]. The observed Glide XP G-scores of −4.033 kcal/mol for BCL-2 and −6.749 kcal/mol for MMP-2 suggest a higher binding affinity for MMP-2 [27,31]. These interactions are further stabilized by specific hydrogen bonds formed with key amino acid residues in the active sites of both proteins [32].

The adsorption properties of SeNPs and EGCG–nano-selenium complexes with BCL2 and MMP2 are based on computational data generated using BIOVIA Materials Studio 2024 [33]. Adsorption energy is a critical parameter in assessing the stability and spontaneity of molecular interactions; a more negative value suggests a more favorable binding process [34]. The MMP2-EGCG-SeNP complex shows a total energy of 1.36 × 10^5^ and a significantly more negative adsorption energy of −1.01 × 10^5^, compared to the MMP2-SeNP system, which has a total energy of 3.64 × 10^4^ and an adsorption energy of −7.69 × 10^4^. The deformation energies are −3.21 × 10^4^ and −3.62 × 10^4^ for the EGCG-SeNP complex and SeNPs alone, respectively. This further supports the conclusion that EGCG enhances the binding and stability of SeNP complex with MMP2 [35]. EGCG is a natural polyphenol with known antioxidative and anticancer properties [36,37]. Its ability to enhance the binding affinity of SeNPs can be attributed to several factors. EGCG can stabilize SeNPs, prevent their aggregation, and increase their bioavailability and bioactivity. For instance, starch microgels and EGCG synergistically stabilize SeNPs, improving their properties [36]. EGCG possesses multiple hydroxyl groups that can participate in hydrogen bonding and other polar interactions with the amino acid residues in the binding sites of BCL2 and MMP2 [38,39]. These additional interactions contribute to a more stable complex [40].

Sleep deprivation is increasingly acknowledged as a major contributor to mood disorders, cognitive impairment, and neuronal dysfunction. Experimental models have shown that prolonged wakefulness induces behavioral deficits resembling depression and anxiety, alongside neurochemical, oxidative, and inflammatory changes that compromise brain integrity [41,42]. The current study was designed to systematically investigate these the pathological consequences of sleep deprivation and to evaluate the protective effects of sodium selenite, epigallocatechin gallate, and their nano-formulation.

Rats subjected to 24 h of sleep deprivation exhibited significant behavioral anomalies, including reduced grooming, rearing, and sucrose intake. These results confirm the presence of anhedonia and reduced exploratory activity, which are characteristic features of depression-like behavior. The present study employed a 24 h sleep deprivation protocol as an established model for inducing acute oxidative and neuro-inflammatory responses in cortical tissues. While this paradigm effectively produces measurable neuronal stress, it may not fully reflect the pathophysiological consequences of chronic or recurrent sleep deprivation observed in humans. Future studies using prolonged or repeated sleep deprivation protocols will be essential to extend the translational value of our findings.

These outcomes are consistent with the notion that sleep loss disrupts motivational and reward-related circuits in the brain, particularly within the prefrontal cortex and limbic system. Reduced sucrose preference reflects anhedonia, while decreased grooming and rearing indicate apathy and diminished exploratory drive. Our findings are in agreement with previous studies showing that rodents exposed to sleep deprivation demonstrate decreased sucrose consumption and reduced locomotor activity, which have been linked to monoaminergic dysregulation and HPA axis hyperactivation [43,44]. Sleep deprivation elevates corticosterone, which perturbs dopaminergic and serotonergic signaling, thereby suppressing reward-related behaviors. The resulting imbalance in neurotransmission contributes to depression-like phenotypes. Co-treatment with sodium selenite, EGCG, and EGCG-SeNPs reversed the behavioral deficits. Notably, EGCG-SeNPs restored grooming, rearing, and sucrose intake to levels comparable to normal controls, suggesting near-complete recovery of motivation and exploratory behavior. Sodium selenite likely exerted its effects through antioxidant and stress-modulating actions, while EGCG acted via anti-inflammatory and neurotransmitter-stabilizing properties. The EGCG-SeNPs combined these benefits with enhanced bioavailability, leading to superior normalization of behavioral outcomes. Sleep-deprived rats showed significant reductions in dopamine, serotonin, norepinephrine, and Grin1 expression, along with increased monoamine oxidase activity. This pattern reflects impaired monoaminergic transmission, a key pathological mechanism underlying depressive-like states. The reduction in Grin1, a glutamatergic NMDA receptor subunit, also indicates disrupted excitatory neurotransmission, contributing to cognitive deficits. These results align with studies reporting that sleep deprivation depletes cortical monoamines and enhances monoamine oxidase activity [45,46]. Similar reductions in serotonin and dopamine have been observed in models of sleep loss, correlating with depressive symptoms [47,48]. Sleep deprivation activates stress pathways, increasing corticosterone, which downregulates monoaminergic systems. The concurrent rise in monoamine oxidase accelerates neurotransmitter degradation, exacerbating neurotransmitter deficits. Sodium selenite, EGCG, and EGCG-SeNPs all increased dopamine, serotonin, norepinephrine, and Grin1, while reducing monoamine oxidase activity. EGCG-SeNPs demonstrated the strongest effect, with neurotransmitter levels even surpassing control values in some cases. Sodium selenite likely enhanced monoaminergic stability via antioxidant protection of neuronal terminals. EGCG inhibited monoamine oxidase and preserved neurotransmitter levels. EGCG-SeNPs combined these actions and provided more efficient neuronal uptake, thus potentiating monoaminergic restoration. Sleep-deprived rats exhibited a significant reduction in cortical acetylcholine and GABA, alongside elevated acetylcholinesterase activity. These changes indicate impaired cholinergic and inhibitory signaling, both of which are essential for learning, memory, and emotional regulation. The decline in acetylcholine contributes to cognitive impairment, while reduced GABA promotes hyperexcitability and anxiety-like behavior. Previous studies demonstrated that sleep deprivation leads to cholinergic dysfunction and diminished GABAergic transmission, which are strongly associated with cognitive decline and mood instability [49,50]. Sleep deprivation disrupts neuronal homeostasis, leading to reduced synthesis and accelerated degradation of acetylcholine, together with downregulation of GABAergic tone. Sodium selenite, EGCG, and EGCG-SeNPs restored acetylcholine and GABA, while reducing acetylcholinesterase activity. EGCG-SeNPs produced the strongest effect, elevating neurotransmitter levels beyond normal controls. Sodium selenite likely acted through redox modulation, protecting cholinergic neurons. EGCG improved neurotransmitter homeostasis via its antioxidant and anti-inflammatory roles. EGCG-SeNPs provided enhanced penetration and bioavailability, resulting in superior restoration of the excitatory–inhibitory balance. Sleep deprivation induced pronounced oxidative stress, evidenced by increased MDA and NO, with decreased Nrf2 expression and reduced GSH, GPx, catalase, and SOD activities. This oxidative imbalance reflects excessive production of ROS and reactive nitrogen species (NOS), coupled with suppression of endogenous antioxidant defenses. Such alterations damage cellular macromolecules and exacerbate neuronal vulnerability. Our findings agree with prior reports that sleep loss enhances oxidative damage in the prefrontal cortex and hippocampus [4,51,52]. Suppression of Nrf2 signaling during sleep deprivation has also been described as a pivotal factor in oxidative injury. All three treatments significantly decreased oxidative markers and enhanced antioxidant defenses, with EGCG-SeNPs providing the most robust effect, including overcompensation above normal control values. Sodium selenite contributes as a cofactor for glutathione peroxidase, enhancing antioxidant activity. EGCG directly scavenges free radicals and activates Nrf2. Although our findings suggest that EGCG-SeNPs may exert their neuroprotective actions through modulation of the NOS2/Nrf2 axis, it should be noted that this interpretation is based on correlative expression data rather than direct mechanistic evidence. Future studies employing targeted inhibition or activation of Nrf2 and NOS2, as well as downstream signaling analysis, will be essential to confirm the causal involvement of this axis in mediating the observed effects. EGCG-SeNPs not only preserved EGCG activity but amplified Nrf2 signaling and downstream antioxidants due to improved bioavailability and sustained release. Sleep deprivation markedly upregulated cortical NOS2, TNF-α, IL-1β, and IL-6, alongside elevated corticosterone levels. This demonstrates strong neuroinflammation and hyperactivation of the hypothalamic–pituitary–adrenal axis, which are major drivers of neuronal dysfunction during sleep loss. Previous studies have consistently reported elevated pro-inflammatory cytokines and corticosterone after sleep deprivation, linking them to depressive behavior and impaired cognition [53,54]. Sodium selenite, EGCG, and EGCG-SeNPs attenuated neuroinflammation and reduced corticosterone levels. EGCG-SeNPs showed superior anti-inflammatory and anti-stress actions, suppressing cytokines below normal control levels in some cases. Sodium selenite modulated immune responses through selenoproteins. EGCG suppressed NF-κB activation and cytokine production. EGCG-SeNPs combined these effects with enhanced cortical delivery, enabling more powerful inhibition of neuroinflammation. Sleep deprivation increased cortical apoptosis, as indicated by higher caspase-3 and Bax and lower Bcl-2. This suggests activation of the intrinsic apoptotic pathway, leading to neuronal loss and structural damage. Previous studies confirmed that sleep loss triggers apoptotic cascades in the prefrontal cortex and hippocampus [55,56,57].

**Figure 16:**
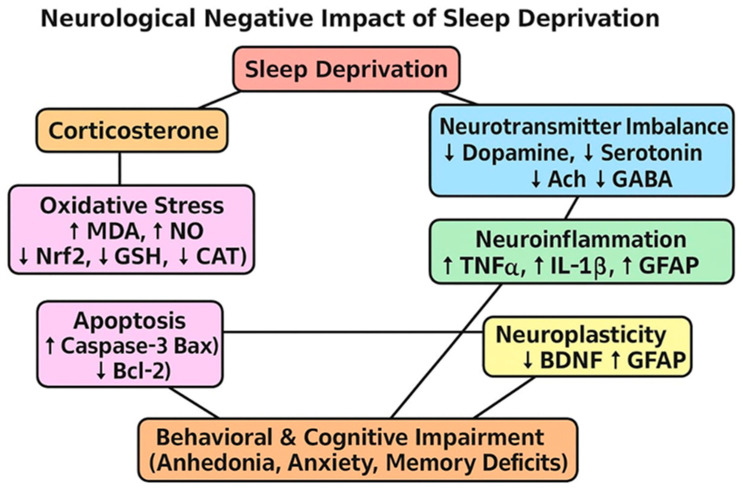
Neurological negative impact of SD.

Our findings imply a mechanistic pathway for neuronal cell death. Sleep deprivation induces oxidative stress, provokes inflammation, and leads to the activation of mitochondrial apoptosis, causing upregulation of Caspase-3, Bax, and reducing Bcl-2, which causes neuronal death as shown in Figure 16.

Sodium selenite, EGCG, and EGCG-SeNPs suppressed apoptosis, reducing caspase-3 and Bax while restoring Bcl-2. EGCG-SeNPs exerted the most pronounced anti-apoptotic effect, normalizing pro- and anti-apoptotic markers. Sodium selenite protected mitochondrial integrity. EGCG inhibited apoptotic signaling via antioxidant and anti-inflammatory pathways. EGCG-SeNPs provided sustained, enhanced delivery that maximized anti-apoptotic potential. Sleep-deprived rats showed reduced BDNF and elevated GFAP, indicating impaired neuroplasticity and astrogliosis. BDNF is critical for neuronal survival and synaptic plasticity; its decline reflects diminished neurotrophic support. GFAP upregulation indicates reactive gliosis, a pathological marker of neuroinflammation. Our findings are consistent with reports of sleep loss suppressing BDNF and inducing GFAP expression [58,59,60].

Sleep deprivation reduced BDNF, leading to impaired synaptic plasticity and memory, causing increase in GFAP which leads to astrogliosis. All treatments increased BDNF and reduced GFAP, with EGCG-SeNPs again showing the strongest effect. Sodium selenite maintained glial antioxidant capacity, while EGCG upregulated BDNF via CREB activation, and EGCG-SeNPs combined these effects with greater bioavailability, leading to enhanced neuronal plasticity [61]. Although EGCG-SeNPs showed significant neuroprotective efficacy in cortical tissues, the present study did not include direct pharmacokinetic evaluation or biodistribution analysis to confirm nanoparticle penetration into the brain. Future investigations employing fluorescent or radiolabeled EGCG-SeNPs will be essential to delineate their brain uptake, tissue accumulation, and pharmacokinetic profile.

**Figure 17:**
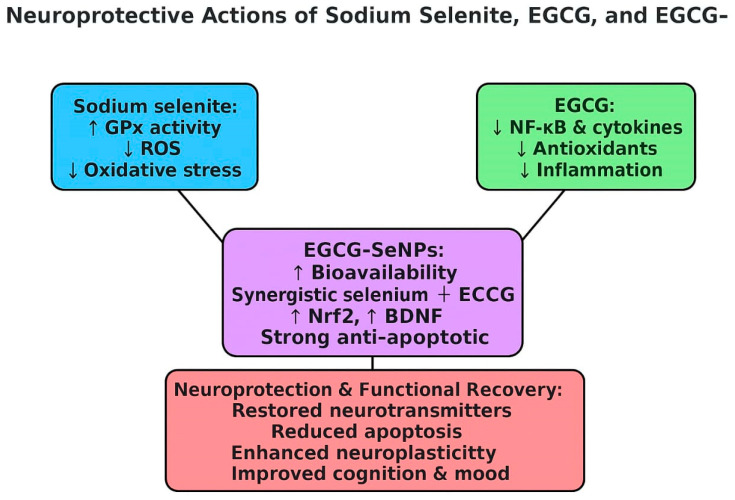
Neuroprotective actions of sodium selenate, EGCG, and EGCG-SeNPs.

Collectively, these results confirm that sodium selenite, EGCG, and EGCG-SeNPs confer significant neuroprotection against sleep deprivation-induced behavioral, biochemical, and structural changes. Among them, EGCG-SeNPs consistently produced the most robust effects, surpassing both sodium selenite and EGCG. This superiority reflects the synergistic activity of selenium and EGCG, together with the improved bioavailability and sustained action provided by the nanoparticle formulation as shown in Figure 17.

It should be noted that the current study employed an acute (24 h) sleep deprivation paradigm, which primarily reflects short-term neuronal and behavioral consequences. While this model effectively induces measurable cortical oxidative and inflammatory changes, it does not fully replicate the cumulative neurodegenerative impact of chronic sleep loss observed in human conditions. Future studies employing extended or repeated sleep deprivation protocols would be valuable to enhance the translational relevance of these findings. While the current study demonstrated apoptotic involvement through alterations in BAX, Bcl-2, and caspase-3 levels, confirmation via TUNEL staining would further strengthen these findings by directly identifying DNA fragmentation in apoptotic neurons. This will be addressed in future investigations.

Interestingly, sodium selenite, EGCG, and EGCG-SeNP administration mitigated cognitive manifestations and cortical histopathological anomalies in the sleep deprivation-subjected rats. These beneficial effects were elicited by sodium selenite, EGCG, and EGCG-SeNPs and may be related to their ability to counteract corticosterone spike, cortical neurotransmitter decrease, and neurotropic and glial marker alterations, while also inhibiting the inflammatory and apoptotic axis and stimulating Nrf2 antioxidant cascade. The present study was limited to an acute rat model of sleep deprivation, which may not fully capture chronic human sleep loss.

## 4. Materials and Methods

### 4.1. In Silico Analysis

#### 4.1.1. Molecular Docking

Protein–ligand docking was executed with Schrödinger Suite 2023-3 Maestro using the Glide module. Human BCL-2 (PDB 4AQ3) and MMP-2 (PDB 1QIB) were prepared with the Protein Preparation Wizard: hydrogens were added, bond orders were assigned, disulfide bonds were created, and water molecules > 5 Å were removed from hetero-atoms; protonation states were predicted by PROPKA at pH 7.4 ± 0.2 and the structures were energy-minimized with the OPLS4 force field (RMSD restraint 0.30 Å). (+)-Epigallocatechin gallate (EGCG) was sketched in 2-D Sketcher, converted to 3-D, desalted, and energetically minimized (LigPrep, OPLS4, 32 stereoisomers, pH 7.0 ± 2.0). Receptor grids (20 Å side length) were centered on the catalytic/active-site clefts. Flexible ligand docking was performed sequentially in Standard Precision (SP) and Extra Precision (XP) modes, retaining up to 10 poses per ligand; final poses were re-scored with Prime MM-GBSA (VSGB 2.0 solvent model). The lowest-energy complexes were inspected for hydrogen bonds, π–π stacking, salt bridges, and metal contacts; Glide XP G-scores are reported.

#### 4.1.2. Absorptivity of Nano-Selenium and EGCG–Nano-Selenium with BCL2 and MMP2

The adsorption properties of nano-selenium and EGCG–nano-selenium with BCL2 and MMP2 structures were investigated using BIOVIA Materials Studio 2024. The lowest-energy poses were initially determined using the Adsorption Locator module, which employs the Forcite and COMPASS III force fields. Subsequently, total energy, adsorption energy, rigid adsorption energy, and deformation energy were calculated for each protein–ligand system to quantify binding stability and conformational changes.

### 4.2. Drugs

EGCG (CAS No. 989-51-5) was obtained from Sigma-Aldrich (St. Louis, MO, USA) and employed without any further purification. The green synthesis of selenium nanoparticles (SeNPs) functionalized with EGCG (SeNPs-EGCG) was achieved by mixing 10 mL of sodium selenite solution (Na_2_SeO_3_, 10 mM) with an equal volume of EGCG solution (3.5 mg/mL). The reaction mixture was maintained under constant magnetic stirring at ambient temperature for 12 h, during which EGCG served simultaneously as a reducing and stabilizing agent to promote nanoparticle formation. The appearance of a characteristic reddish coloration signified successful synthesis of SeNPs-EGCG. The colloidal suspension was subsequently lyophilized using a laboratory freeze-drying system (FreeZone 4.5 L, Labconco, Marshall Scientific, Hampton, NH, USA), and the resulting dry nanoparticle powder was collected for use in downstream experimental applications.

#### 4.2.1. Characterization of Nanoparticle

##### Zeta Potential and Particle Size Distribution

The physicochemical characteristics of the synthesized selenium nanoparticles (SeNPs) were evaluated using a Zetasizer Nano ZS90 (Malvern Panalytical Ltd., Malvern, Worcestershire, UK) at Helwan University to determine their hydrodynamic size distribution and surface charge. Measurements were performed in ultrapure water at 25 °C using a clear disposable zeta cell, with prior sonication to minimize aggregation [62,63]. Dynamic light scattering (DLS) analysis indicated size distribution, with intensity broad peaks observed at ~18–20 nm and another very narrow peak at ~80–90 nm. The zeta potential analysis revealed a mean surface charge of −48.0 mV, with three distinct peaks at −51.1 mV (55.1%), −28.8 mV (25.0%), and −86.3 mV (19.9%). The strongly negative overall zeta potential reflects good colloidal stability, driven by electrostatic repulsion between particles [64,65].

##### Transmission Electron Microscopy (TEM)

The morphology and ultrastructural features of the EGCG-mediated SeNPs were further examined by high-resolution-TEM (TEM; JEOL Ltd., Mitaka, Tokyo, Japan) at Al-Azhar University. For sample preparation, a small aliquot of the aqueous nanoparticle suspension was sonicated to disrupt potential aggregates, then drop-cast onto carbon-coated copper grids. Excess liquid was gently blotted with filter paper, and the grids were left to dry under ambient conditions prior to imaging [66,67].

TEM were acquired at optimized accelerating voltages, providing high-contrast visualization of the nanoparticles at nanometer resolution. The images confirmed the nanoscale integrity of the synthesized particles, as well as their shape, size, and spatial distribution within the colloidal system.

##### UV–Visible Spectroscopic Analysis

The optical properties of the green-synthesized EGCG-mediated selenium nanoparticles (EGCG-SeNPs) were characterized through ultraviolet–visible (UV–Vis) spectroscopy using a Shimadzu spectrophotometer (Shimadzu Corporation, Kyoto, Japan) at Badr University in Cairo, Egypt. The absorption spectra were recorded over the wavelength range of 200–800 nm to monitor the surface plasmon resonance (SPR) signature, confirming the successful synthesis of selenium nanoparticles.

##### Methods of FTIR Analysis

Fourier transform infrared (FTIR) spectroscopy was employed to identify the characteristic functional groups of pure Epigallocatechin gallate (EGCG) and to investigate its interaction with nano-selenium in the EGCG-SeNP complex. The spectra were recorded in the wavenumber range of 4000 to 400 cm^−1^. For the analysis, samples were prepared using the conventional KBr pellet method, where a small amount of the sample was thoroughly mixed with dry potassium bromide (KBr) and compressed into a thin, transparent pellet. The instrument was set to a resolution of 4 cm^−1^, and an average of 32 scans was performed for each spectrum to ensure a high signal-to-noise ratio. The resulting spectra were plotted as transmittance (%) versus wavenumber (cm^−1^), and the peak positions were determined and assigned to their corresponding molecular vibrations [68].

### 4.3. Experimental Design

#### 4.3.1. Experimental Animals

Forty healthy adult male albino rats from Badr University animal house weighing between 120 and 130 g were employed for this investigation. Animals were housed in polycarbonate cages with stainless steel wire lids, accommodating no more than five rats per cage, and wood shavings were provided as bedding material. Environmental conditions were carefully regulated, including a 12:12 h light–dark cycle, maintenance of room temperature at 22 ± 3 °C, and controlled relative humidity. Standard pelleted chow and drinking water were supplied ad libitum. Prior to initiation of the experimental procedures, animals were allowed a two-week acclimatization period to adapt to the laboratory environment. All housing, handling, and experimental protocols conformed to internationally accepted guidelines for the care and use of laboratory animals and received prior approval from the Ethics Committee of School of Biotechnology, Badr University in Cairo, Egypt (Approval No. BUC-IACUC/BIOT/009/KM/2025).

#### 4.3.2. Experimental Grouping

It involves 40 animals in five distinct groups, with 8 animals per group subjected to specific conditions and treatments. The animals were grouped as following: (Group 1) (CNT) animals of this group were exposed to standard conditions and injected with saline orally for 8 days contentiously; (Group 2) (24 hrSD) animals of this group were injected with saline orally for 8 days contentiously, and on day 7 the animals were exposed to 24 h of sleep deprivation; (Group 3) (24 hrSD/Na_2_SeO_3_) animals of this group was injected orally with Na_2_SeO_3_, with 0.5 mg/kg/day for 8 days continuously, and on day 7 animals were exposed to 24 h of sleep deprivation [69]; (Group 4) (24 hrSD/EGCG) animals of this group was injected orally with EGCG, with 100 mg/kg/day for 8 days continuously, and on day 7 animals were exposed to 24 h of sleep deprivation [70,71]; (Group 5) (24 hrSD/EGCG-SeNPs) animals of this group were injected orally with selenium nanoparticles biosynthesized using EGCG, with 0.5 mg/kg/day for 8 days continuously, and on day 7 animals were exposed to 24 h of sleep deprivation [72].

### 4.4. Sleep Deprivation Induced by Rapid Eye Movement (REM) Sleep Interruption

Paradoxical sleep-deprived groups were subjected to the modified multiple platform method, in which animals were placed on small platforms surrounded by water. In this setup, the natural fear of sleep (REM sleep) and a loss of postural muscle tone (atonia) occurred, causing the animals to lose balance and fall into the water. This interruption effectively prevented the continuation of REM sleep. Throughout the deprivation period, all animals had full access to food and water [73].

### 4.5. Behavioral Assessments

To investigate behavioral alterations relevant to depression-like states, two well-established paradigms were utilized. The Open-Field Test (OFT) served to evaluate locomotor activity and exploratory behavior, as well as indices of anxiety. Each rat was gently placed at the center of a transparent plexiglass arena, and behavioral parameters including total distance traveled, frequency of rearing, and time spent in the central zone were systematically recorded and analyzed [74,75].

In parallel, the sucrose preference test (SPT) was employed to assess anhedonia, a hallmark behavioral correlate of depressive phenotypes. Animals were initially habituated to two identical drinking bottles, with one containing 1% sucrose solution and the other plain water, to minimize neophobic responses. Subsequently, rats were allowed unrestricted access to both solutions for 24 h. Sucrose preference was represented as the percentage of sucrose solution drank relative to total fluid intake. This provided a quantitative assessment of reward sensitivity and hedonic drive [76]. OFT and SPT were selected to evaluate exploratory behavior, locomotor activity, and anhedonia, which are key indicators of affective and motivational disturbances associated with acute sleep deprivation.

### 4.6. Scarification and Tissue Collection

Following the completion of all behavioral evaluations, animals were humanely euthanized by rapid decapitation, which was a method selected to minimize procedural stress and ensure immediate preservation of neural tissue integrity. Firstly, as fast as possible, the blood was collected and then centrifuged for 10 min at 5000 rpm to obtain highly clear serum used for hormonal parameters. Also, the brains were carefully excised without delay, gently blotted to remove excess moisture, and handled with caution to prevent mechanical artifacts. Each prefrontal cortex of the brain was subsequently divided into two major portions. One portion was fixed in formalin and long-term preservation, intended for subsequent histopathological assessments. The other portion of the prefrontal cortex of the brain tissues was homogenized in PBs buffer (pH 7.4). The homogenate was centrifuged at 5000 rpm and 4 °C for 10 min, and the supernatant was kept at −80 °C until analysis. This supernatant was utilized to determine oxidative stress, inflammation, apoptosis, gene expression, neurotransmitters, and enzyme characteristics.

### 4.7. Biochemical Assays

Biochemical investigations were primarily performed on homogenates prepared from the prefrontal cortex, with the exception of corticosterone, which was quantified in serum samples using ELISA and commercially available ELISA kits (Dynatech Microplate Reader Model MR 5000, Santa Monica, CA, USA) according to the manufacturers’ instructions.

#### 4.7.1. Evaluation of Oxidative and Antioxidant Level

Indices of oxidative stress were determined through multiple biochemical endpoints. Lipid peroxidation (LPO) was estimated by measuring MDA content, a reliable indicator of membrane lipid damage [77]. NO concentration was assessed spectrophotometrically at 540 nm [78]. Reduced GSH, representing a key intracellular antioxidant and redox regulator, was quantified using the classical Ellman’s method [79]. To further assess the antioxidant defense system, enzymatic activities of SOD [80], CAT, GPx [81], as well as Nrf2, a transcription factor regulating antioxidant gene expression, were quantified to provide molecular insight into redox homeostasis Table 5.

#### 4.7.2. Inflammatory Mediators

Neuro-inflammatory status was evaluated by measuring TNF-α, IL-6, and IL-1β, using ELISA kits [82,83]. Expression of inducible Nos2, a marker of nitrosative stress and inflammation, was determined at the molecular level (Table 5).

#### 4.7.3. Apoptotic Markers

To evaluate neuronal apoptosis, the expression levels of Bax and caspase-3, as well as Bcl-2, were quantified. These markers collectively provided insight into the balance between pro-death and pro-survival pathways under experimental conditions using specific ELISA kits [84].

#### 4.7.4. Neurotransmitter and Enzymatic Activity

Levels of monoamine neurotransmitters, including serotonin, dopamine, and norepinephrine, were measured in prefrontal cortex homogenates. Cholinergic function was evaluated by quantifying acetylcholine levels alongside acetylcholinesterase (AChE) activity. Monoamine oxidase (MAO) activity, responsible for monoamine degradation, was also determined. Expression of glutamate receptor ionotropic NMDA subunit 1 (Grin1) was analyzed as a molecular marker of excitatory neurotransmission (Table 5) [85].

#### 4.7.5. Neurotrophic and Glial Markers

BDNF was measured as an index of synaptic plasticity and neuronal survival using an ELISA kit sourced from My Bio-Source (Cat. No. MBS355435; San Diego, CA, USA). Assays were performed according to the supplier’s technical instructions, ensuring optimal specificity and sensitivity. Glial fibrillary acidic protein (GFAP) levels were assayed to assess astrocytic activation and gliosis using the commercially available ELISA kit (Cat. No. NS830; Merck, Darmstadt, Germany), following the manufacturer’s validated protocol [86].

#### 4.7.6. Stress Hormone

Serum corticosterone, a key indicator of HPA axis activity, was quantified by ELISA as a biomarker of physiological stress response.

### 4.8. Gene Expression

RNA was extracted utilizing RNeasy Plus Minikit from tissues from all groups. RevertAid™ H Minus Reverse Transcriptase was used for complementary DNA (cDNA) synthesis. qRT-PCR was performed utilizing the BioRad SYBR green PCR Master Mix kit (Life Technologies, Carlsbad, CA, USA), in which the Rotor-Gene 6000 instrument (QIAGEN, Hilden, Germany) was employed. Nrf2, Nos2, and Grin1, with β-actin used as control were assessed (Table 5). The typical thermal profile is 95 °C for 4 min, followed by 40 cycles of 94 °C for 60 s and 55 °C for 60 s. The fold differences in gene expression between the control and treatment groups by calculation of 2^−ΔΔCt^ was calculated [87,88].

### 4.9. Immunohistochemical Analysis

We used immunohistochemistry (IHC) to investigate the expression and location of GFAP and NF-κB in the prefrontal cortex sections. As part of these treatments, sections were processed and antigens were retrieved by boiling slides in citrate buffer. Hydrogen peroxide (3%) was administered for 20 min to inhibit tissue endogenous peroxidase enzyme activity. Then, 5% bovine serum albumin was utilized as a blocking reagent to prevent non-specific antibodies from linking to tissue proteins. A total of 4 µm of prefrontal cortex samples were stained with GFAP and NF-κB primary antibodies for 90 min [89]. The obtained specimens were then treated for 30 min with HRP-labeled secondary antibodies. Then, the diaminobenzidine (DAB) kit (ScyTek Laboratories, Inc., Logan, UT, USA) was applied, and hematoxylin was utilized as a counterstain. Images were acquired with a digital imaging equipment coupled to a light microscope (Leica DM 500 with Flexacam i5, Wetzlar, Germany). GFAP and NF-κB immunoreaction area percentages were assessed in all groups at ×400 magnification [90].

### 4.10. Quantitative Assessment of IHC Staining

This step is an effective tool for studying protein location inside the tissue. Semi-quantitative IHC is conducted using the ImageJ Fiji software, version 1.2. Deconvolution and downstream examination were performed. GFAP and NF-κB immunoreaction area percentage was elucidated at magnification of X 400 for all groups [91].

### 4.11. Histopathological Examination

Prefrontal cortex specimens fixed in 10% of formalin solution underwent histological examination through staining with hematoxylin and eosin (H&E) [92]. Stained sections were examined under a light microscope to evaluate neuronal morphology, cytoarchitecture, and evidence of degenerative alterations. This assessment facilitated the identification of neuropathological changes induced by sleep deprivation and the extent of neuroprotection afforded by the treatment regimens.

### 4.12. Statistical Analysis

All data were expressed as mean ± standard error of the mean (SEM). Statistical analyses were performed using one way analysis of variance (ANOVA), followed by Tukey’s multiple comparison post hoc test to evaluate intergroup differences. Analyses were conducted using the GraphPad Prism software (version 9.0; GraphPad Software, San Diego, CA, USA). A probability value of *p* < 0.05 was considered statistically significant.

## 5. Conclusions

This study demonstrates that eco-synthesized EGCG-SeNPs effectively counteract the detrimental effects of sleep deprivation by restoring neurotransmitter balance, reinforcing antioxidant defenses, suppressing neuroinflammation, inhibiting apoptosis, and enhancing neurotrophic support. These multifaceted actions translated into marked improvements in behavior and cortical integrity. Importantly, EGCG-SeNPs outperformed sodium selenite and EGCG, highlighting nanotechnology as a powerful strategy for maximizing the therapeutic efficacy of phytochemicals and trace elements.

## Figures and Tables

**Table 1:** Molecular docking results of EGCG with anti-apoptotic BCL-2 and Metalloproteinase MMP-2.

Target Protein	Glide XP G-Score (kcal mol^−1^)	Binding Affinity Classification	Hydrogen-Bonding Residues
Anti-apoptotic BCL-2	−4.033	Good	Leu 121, Glu 160, Asn 163, Arg 164
Metalloproteinase MMP-2	−6.749	High	Phe 80, Pro 466, Gly 505, Pro 506

**Table 2:** Comparative adsorption energetics of nano-selenium and EGCG-functionalized nano-selenium adsorbed on BCL-2 and MMP-2.

Structures	Ligand	Total Energy	Adsorption Energy	Rigid Adsorption Energy	Deformation Energy	MMP2 (2): dEad/dNi	dEad/dNi
BCL2	SeNPs	2.49 × 10^4^	−5.44 × 10^4^	2.467	−5.44 × 10^4^	−1.19 × 10^4^	−4.25 × 10^4^
	EGCG-SeNPs	1.21 × 10^5^	−8.16 × 10^4^	6.015	−8.16 × 10^4^	−1.026 × 10^4^	−7.14 × 10^4^
MMP2-1	SeNPs	3.64 × 10^4^	−7.69 × 10^4^	−22.15	−7.68 × 10^4^	−3.62 × 10^4^	−4.07 × 10^4^
	EGCG-SeNPs	1.36 × 10^5^	−1.01 × 10^5^	−8.16	−1.01 × 10^5^	−3.21 × 10^4^	−6.91 × 10^4^

**Table 3:** FTIR peak assignments for pure EGCG.

Wavenumber (cm^−1^)	Assignment (Functional Group)
3241	O-H stretching (Phenolic, H-bonded)
2918, 2849	C-H stretching (Aliphatic-CH_2_, -CH_3_)
1704	C=O stretching (Ester carbonyl)
1610, 1448	C=C stretching (Aromatic ring)
1242, 1053	C-O stretching (Phenolic and ester)

**Table 4:** Comparison of FTIR peaks between EGCG and EGCG-SeNPs.

Wavenumber (cm^−1^) (EGCG)	Wavenumber (cm^−1^) (EGCG-SeNPs)	Change Observed	Interpretation
3241	~3225	Shift to lower wavenumber, broadening	Involvement of phenolic-OH in coordination/interaction with nano-Se.
2918, 2849	2916, 2847	Minor shift	Aliphatic C-H groups are not directly involved in the interaction.
1704	1698	Shift to lower wavenumber	Coordination of ester carbonyl oxygen with nano-Se surface.
1610, 1448	1608, 1450	Minor shift	Slight change in the electronic environment of the aromatic rings.
1242, 1053	1235, 1048	Shift to lower wavenumber	Involvement of C-O groups (phenolic/ester) in binding to nano-Se.

**Table 5:** Primer sequences of target genes analyzed by qRT-PCR, including Nrf2, Nos2, and Grin1, with β-actin used as the internal control.

	Forward Sequence	Reverse Sequence	Accession Number
*Nrf2*	CCTCAGCATGATGGACTTGGA	GCGACTGAAATGTAGGTGAAGA	NM_001399173.1
*Nos2*	GGTGAGGGGACTGGACTTTTAG	TTGTTGGGCTGGGAATAGCA	NM_012611.3
*Grin1*	GGCAACTTGTATGGGAGCCT	GGCAACTTGTATGGGAGCCT	NM_012573.4
*Β-actin*	GTCCACCCGCGAGTACAAC	GGATGCCTCTCTTGCTCTGG	NM_031144.3

## Data Availability

The original contributions presented in this study are included in the article. Further inquiries can be directed to the corresponding authors.

## Abbreviations

The following abbreviations are used in this manuscript:

SD	Sleep deprivation
EGCG	Epigallocatechin-3-gallate
Bcl2	B-cell lymphoma-2
MMP2	Matrix metallopeptidase 2
SeNPs	Selenium nanoparticles
Nrf2	Nuclear factor erythroid 2-related factor 2
NOS2	Nitric Oxide Synthase 2
MDA	Malondialdehyde
LPO	Lipid peroxidation
NO	Nitroxide
GSH	Glutathione synthetase
SOD	Superoxide dismutase
CAT	Catalase
TNF-α	Tumor necrosis factor alpha
IL-6	Interlukin-6
IL-1β	Interlukin-1β
Bax	Bcl-2 Associated X-protein
5-HT	Serotonin
DA	Dopamine
NE	Norepinephrine
Ach	Acetylcholine
AChE	Acetylcholinesterase
MAO	Monoamine Oxidase
BDNF	Brain-derived neurotrophic factor
GFAP	Glial fibrillary acidic protein
HPA	Hypothalamic–pituitary–adrenal
TEM	Transmission electron microscopy
GABA	γ-aminobutyric acid
SEM	Standard error of the mean
GPx	Glutathione peroxidase
Grin1	Glutamate receptor ionotropic NMDA subunit 1
NMDA	N-methyl-D-aspartate
PDB	Protein data bank
OFT	Open-Field Test
SPT	Sucrose Preference Test
cDNA	Complementary DNA
